# Green Tea Extract Supplementation Induces the Lipolytic Pathway, Attenuates Obesity, and Reduces Low-Grade Inflammation in Mice Fed a High-Fat Diet

**DOI:** 10.1155/2013/635470

**Published:** 2013-01-29

**Authors:** Cláudio A. Cunha, Fábio S. Lira, José C. Rosa Neto, Gustavo D. Pimentel, Gabriel I. H. Souza, Camila Morais Gonçalves da Silva, Cláudio T. de Souza, Eliane B. Ribeiro, Alexandra Christine Helena Frankland Sawaya, Cláudia M. Oller do Nascimento, Bruno Rodrigues, Patrícia de Oliveira Carvalho, Lila M. Oyama

**Affiliations:** ^1^Departamento de Fisiologia, Disciplina de Fisiologia da Nutrição, Universidade Federal de São Paulo (UNIFESP), 04023-060 São Paulo, SP, Brazil; ^2^Laboratory of Exercise Biochemistry and Physiology, Health Sciences Unit, University of Southern Santa Catarina, 88806-000 Criciúma, SC, Brazil; ^3^Department of Internal Medicine, State University of Campinas, 13083-887 Campinas, SP, Brazil; ^4^Laboratory of Multidisciplinary Research, São Francisco University (USF), 12916-900 Bragança Paulista, SP, Brazil; ^5^Department of Plant Biology, Institute of Biology, State University of Campinas (UNICAMP), 13083-970 Campinas, SP, Brazil; ^6^Human Moviment Laboratory, São Judas Tadeu University, 05503-001 São Paulo, SP, Brazil

## Abstract

The aim of this study was to evaluate the effects of green tea *Camellia sinensis *extract on proinflammatory molecules and lipolytic protein levels in adipose tissue of diet-induced obese mice. Animals were randomized into four groups: CW (chow diet and water); CG (chow diet and water + green tea extract); HW (high-fat diet and water); HG (high-fat diet and water + green tea extract). The mice were fed *ad libitum *with chow or high-fat diet and concomitantly supplemented (oral gavage) with 400 mg/kg body weight/day of green tea extract (CG and HG, resp.). The treatments were performed for eight weeks. UPLC showed that in 10 mg/mL green tea extract, there were 15 ***μ***g/mg epigallocatechin, 95 ***μ***g/mg epigallocatechin gallate, 20.8 ***μ***g/mg epicatechin gallate, and 4.9 ***μ***g/mg gallocatechin gallate. Green tea administered concomitantly with a high-fat diet increased HSL, ABHD5, and perilipin in mesenteric adipose tissue, and this was associated with reduced body weight and adipose tissue gain. Further, we observed that green tea supplementation reduced inflammatory cytokine TNF**α** levels, as well as TLR4, MYD88, and TRAF6 proinflammatory signalling. Our results show that green tea increases the lipolytic pathway and reduces adipose tissue, and this may explain the attenuation of low-grade inflammation in obese mice.

## 1. Introduction

Obesity is a serious health problem in developed countries, and the prevalence of obesity has increased dramatically for several decades. Both genetic and environmental factors are implicated in the development of obesity, in particular food overconsumption. Being severely overweight or obese is associated with major health risks such as cardiovascular disease, diabetes, nonalcoholic fatty liver disease, and cancer [1]. An important feature of obesity is its association with chronic low-grade inflammation. Adipose tissue is the largest endocrine organ in the body and is characterised by cytokine and chemokine production and acute-phase inflammatory signalling [2–4]. As an established in 3T3-L1 adipocyte cells comprise main features relating to innate immunity [5]; this stage also is known to involve the toll-like receptors (TLRs) [6–9]. Stimulation of TLRs causes an immediate defensive response, including the production of an array of antimicrobial peptides and cytokines [10]; this response includes adaptor molecules, such as myeloid differentiation primary response gene 88 (MyD88) and the tumour necrosis factor receptor-associated factor 6 (TRAF6) [11]. 

The adipose tissue is involved in metabolic, physiological, and immunological regulation including the cytokines. Adipose tissue fat stores are mainly dependent upon fatty acid (FA) supply, FA esterification to triglycerides (TG), and TG breakdown, or lipolysis. Adipose triglyceride lipase (ATGL) and hormone-sensitive lipase (HSL) both have the capacity to degrade TG by cleaving the ester bond, thus governing the lipolysis pathway in adipose tissue [12]. Adipose tissue lipolysis has received much attention over the past 10 years because of its altered regulation in obesity. Therefore, prevention and treatment of obesity should focus on anti-inflammatory effects, and various treatments have emerged, including phytoterapic therapy.

Green tea contains high levels of polyphenols, which may have a number of positive health effects in the prevention of lifestyle-related diseases [13]. Tea is one of the most popular beverages worldwide. Habitual consumption of green tea (*Camellia sinensis*), a popular beverage used in traditional Chinese medicine, has been associated with decreased risks for obesity [14], diabetes [15], hypertension [16], dyslipidemia [17], and CVD mortality [18] in several epidemiological studies. In selected clinical trials, green tea supplementation has been shown to significantly improve features of metabolic syndrome, such as decreased abdominal adiposity indicated by waist circumference in obese subjects [19]. 

Tea and tea components have been reported to possess various biological and pharmacological effects, such as antibacterial actions [20] and lowering plasma lipids and glucose levels [21, 22]. Green tea catechins are efficacious in cell and animal models of obesity, and the proposed modes of action include: decreased adipocyte differentiation and lipogenesis; increased beta-oxidation; and decreased lipid absorption [23]. However, relatively little is known about the underlying mechanism of action, in the regulation of body weight, lipolytic action and its relationship with inflammatory status. The aim of this study was to examine the effects of green tea extract on the body fat mass and lipolytic enzymes in adipose tissue of mice fed a high-fat diet and to observe whether reduction of fat mass is associated with diminished low-grade inflammation.

## 2. Experimental Methods

### 2.1. Animal, Diet, and Green Tea Supplementation

The Experimental Research Committee of the São Paulo Federal University approved (no. 1673/07) all procedures and the care of the animals used in this study. A total of 24 male Swiss mice ranging in age from 8 weeks were used. They were housed four per cage, receiving a chow diet and water *ad libitum*, in an animal room under a 12 h light-dark cycle, at 22 ± 1°C and 60 ± 5% humidity. After the acclimatisation period (1 week), the animals were randomly divided into four groups: (1) control mice (CW) fed on chow diet and placebo supplementation (0.1 mL water/day); (2) (CG) chow diet and green tea supplementation (0.1 mL water + 400 mg green tea extract per kg body weight/day); (3) (HW) a high-fat diet and placebo supplementation (0.1 mL water/day) for 2 months; (4) (HG) high-fat diet and green tea supplementation (0.1 mL water + 400 mg green tea commercial extract per kg body weight/day). The fatty acid composition of chow or high-fat diet diets is detailed in previous study from our group (Table 1) [24]. 

### 2.2. Composition of Green Tea by Ultra-Performance Liquid Chromatography (UPLC)

We evaluated composition of green tea commercial extract by Ultra-performance Liquid Chromatography- Mass Spectrometry. An Acquity UPLC system (Waters, Milford, MA, USA) consisting of a binary solvent manager and a sample manager was coupled to an Acquity TQD Mass Spectrometer (Micromass Waters, Milford, MA, USA). Analyses were performed on a bridged ethylene hybrid (BEH) C18 analytical column (50 mm × 2.1 mm, 1.7 *μ*m, at a temperature of 25°C, injecting 5 *μ*L of extract and standards. A gradient was applied at a flow rate of 0.2 mL min^−1^ using two mobile phases—(A) purified water with 0.1% formic acid; and (B) methanol—starting with 5% B, ramping to 100% B in 8 min, maintained until 8.50 min, returning to the initial conditions. Detection was carried out in the negative ion mode with an ESI source under the following conditions: capillary −3000 V, cone −30 volts, temperature 150°C; ranging between m/z 100–1000. Data acquisition was carried out by MassLynx software. Our data showed that, in Green tea extract, there were 15 *μ*g/mg epigallocatechin, 95 *μ*g/mg epigallocatechin gallate, 20.8 *μ*g/mg epicatechin gallate, and 4.9 *μ*g/mg gallocatechin gallate.

### 2.3. Biochemical Measurements

Eighteen hours after the last oral gavage of green tea extract and after a 12-hour fast, the animals were decapitated, blood was collected, and serum samples were collected after allowing the blood to clot on ice. Serum was stored frozen at −80°C for analysis. Lab Test Kits were used to assess fasting total cholesterol, high-density lipoprotein (HDL-c), and triacylglycerol (TG) levels. The samples were analysed using an enzymatic method. LDL-c and VLDL-c were calculated according to the Friedewald equation ((LDL-c = total cholesterol-(HDL-c)-(TG/5)) and (VLDL = TG/5)) [25]. The Zen-Bio Kit was used to assess free fatty acid.

### 2.4. TNF-*α*, Adiponectin, and IL-10 Protein Level Determination by ELISA

Following decapitation, mesenteric adipose tissue was removed, dissected, homogenised, and centrifuged at 12,000 g for 40 min at 4°C; the supernatant was saved, and the protein concentration was determined using the BCA assay (Bio-Rad, Hercules, California) with bovine serum albumin (BSA) as a reference. Quantitative assessment of adiponectin, TNF-*α*, and IL-10 proteins was carried out by ELISA (DuoSet ELISA, R and D Systems, Minneapolis, MN) following the recommendations of the manufacturer. All samples were run as duplicates, and the mean value is reported.

### 2.5. Protein Analysis by Western Blotting

After euthanasia, the epididymal, retroperitoneal, and mesenteric adipose tissue was dissected and weighed. Mesenteric adipose tissue was homogenised in 1.0 mL solubilisation buffer at 4°C (1% Triton X-100, 100 mM Tris-HCl (pH 7.4), 100 mM sodium pyrophosphate, 100 mM sodium fluoride, 10 mM EDTA, 10 mM sodium orthovanadate, 2.0 mM phenylmethylsulphonyl fluoride (PMSF), and 0.1 mg aprotinin/mL) with a Polytron (model 713T; Fisatom Equipamentos Científicos, São Paulo, SP, Brazil). Insoluble material was removed by centrifugation for 30 min at 9,000 g in a 70.Ti rotor (Beckman, Fullerton, CA, USA) at 4°C. The protein concentration of the supernatants was measured by the BCA assay. Proteins were denatured by boiling (5 min) in Laemmli sample buffer [26] containing 100 mM DTT, run on 8, 10, or 12% SDS-PAGE gels in a Bio-Rad miniature slab gel apparatus. The electrotransfer of proteins from gels to nitrocellulose membranes was performed for ~1.30 h/4 gels at 15 V (constant) in a Bio-Rad semidry transfer apparatus. Nonspecific protein binding to the nitrocellulose was reduced by preincubation for 2 h at 22°C in blocking buffer (1% bovine serum albumine, 10 mM Tris, 150 mM NaCl, and 0.02% Tween 20). The nitrocellulose membranes were incubated overnight at 4°C with antibodies against TLR4, myeloid differentiation primary response gene (88) (MyD88), TNF receptor associated factor (TRAF6), hormone sensitive lipase (HSL), adipose triglyceride lipase (ATGL), comparative gene identification-58 (CGI-58 or ABHD5), perilipin A, and alpha-tubulin obtained from Santa Cruz Biotechnology (Santa Cruz, CA, USA) diluted 1 : 1000 with blocking buffer supplemented with 1% BSA and then washed for 30 min in blocking buffer without BSA. The blots were subsequently incubated with peroxidase-conjugated secondary antibody for 1 h at 22°C. For evaluation of protein loading, membranes were stripped and reblotted with an anti-alpha-tubulin antibody as appropriate. Specific bands were detected by chemiluminescence, and visualisation/capture was performed by exposure of the membranes to RX films. Band intensities were quantified by optical densitometry of developed autoradiographs (Scion Image software-Scion Corporation, Frederick, MD, USA).

### 2.6. Statistical Analysis

The statistical analysis was performed using the GraphPad Prism statistics software package version 5.0 for Windows (GraphPad Software, San Diego, CA, USA). The data are expressed as the means ± SEM. Implementation of the Kolmogorov-Smirnov test revealed that the results of experiments were distributed normally. The data were analysed using ANOVA two ways for comparison between four groups. A value of *P* < 0.05 was considered statistically significant.

## 3. Results

### 3.1. Body Mass and Tissue Weight

The relative weight (tissue weight/total body weight) of epididymal adipose tissue was increased in group HW compared to the GW group and decreased in the HG group compared to the HW group. The mesenteric adipose tissue showed an increase in group HW compared to the GW group and decrease in the HG group compared to the HW group. In retroperitoneal adipose tissue, only the CG group decreased compared with the GW group. Liver and gastrocnemius tissues showed no significant difference between groups (Table 2).

### 3.2. Lipid Profile and Serum Adiponectin

Serum triglycerides and total cholesterol did not differ between any of the groups. The serum concentration of LDL in the HW group was increased compared to the GW group. The concentration of serum HDL in the HG group showed an increase compared to the HW group. The serum concentration of FFA did not differ between any of the four groups. The serum adiponectin in CG group increased compared to the GW group, and the HG group increased compared to the HW group (Table 3).

### 3.3. Cytokines in the Adipose Tissue

The cytokine concentration of adiponectin in the mesenteric adipose tissue in the CG group increased compared to the GW group, and the HG group increased compared to the HW group. The content of IL-10 showed a significant increase in group CG compared to the GW group. The TNF-*α* levels in the mesenteric adipose pad of the HW group showed a significant increase compared to the CW group. However, supplementation of green tea decreased this effect (HG versus HW groups; Table 4).

### 3.4. Quantification of Lipolytic Proteins

**Figure 1:**
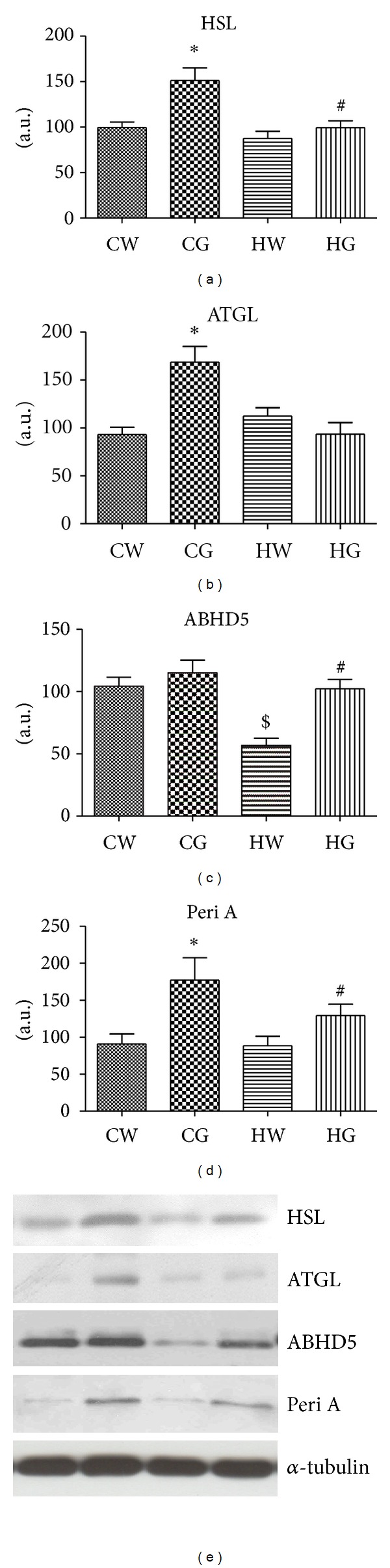
Protein levels of HSL, ATGL, ABHD5, and perilipin A. Mesenteric adipose tissue extracts were immunoblotted with anti-HSL (a), anti-ATGL (b), anti-ABHD5 (c), and Peri A (d). The results of scanning densitometry are expressed as arbitrary units. Bars represent means ± SEM of *n* = 6 mice, *P < 0.05 chow and green tea (CG) group versus chow and water (CW) group, ^$^ *P* < 0.05 high-fat and water (HW) group versus CW group, and ^#^ *P* < 0.05 high-fat and green tea (HG) group versus HW group. In (e), the representative bands of the molecules are shown. The membrane was stripped and immunoblotted with anti-*α*-tubulin antibody and used as loaded protein (lower panel in (e)).

The LSH protein levels showed an increase in mice fed with chow diet supplemented with green tea (CG group) compared to no supplementation (CW group). No difference was observed between the chow and high-fat diet without supplementation. However, there were increased HSL protein levels in the HG group (green tea supplemented) compared to the HW group (*P* < 0.05; Figure 1(a)). The ATGL protein levels only showed an increase in the mice fed with chow diet and supplemented with green tea (Figure 1(b)). In the HW group, the ABHD5 (or CGI-58) protein levels were reduced when compared to the GW group. However, supplementation with green tea strikingly increased the ABHD5 protein levels in obese mice (HG group) when compared to the HW group. No significant difference was observed in the chow-diet groups (Figure 1(c)). The perilipin protein levels increased in mice fed with chow diet supplemented with green tea (CG group) compared to no supplementation (CW group). No difference was observed between the chow and high-fat diets without supplementation. However, green tea increased perilipin protein levels in the HG group compared to the HW group (Figure 1(d)).

### 3.5. Quantification of Inflammatory Proteins

**Figure 2:**
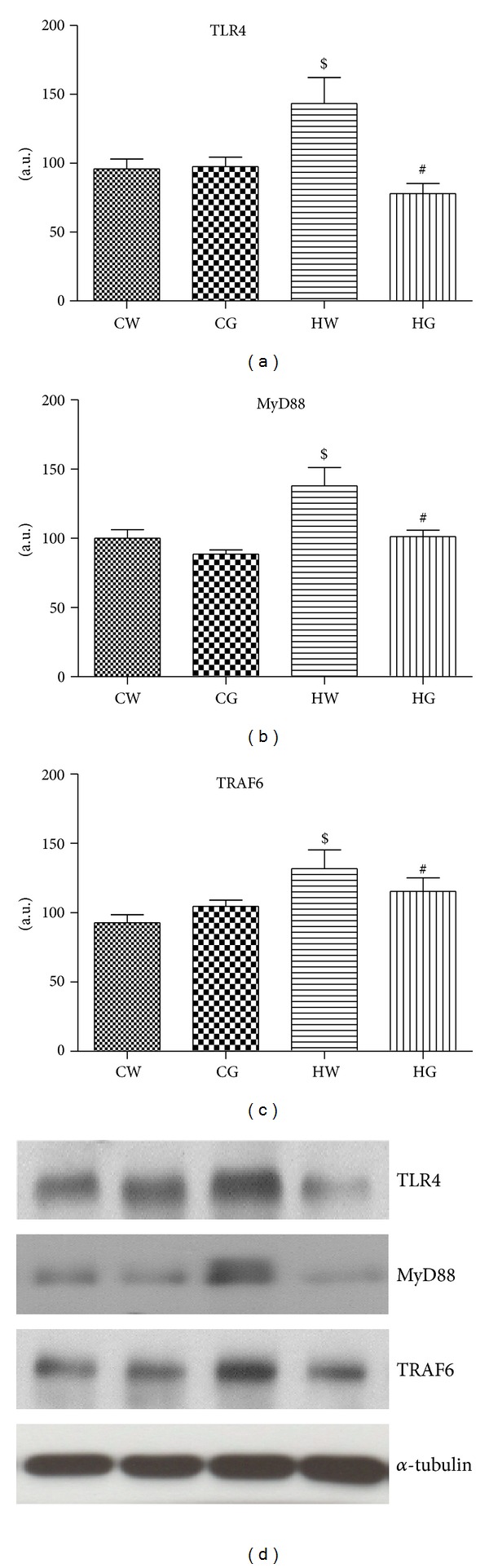
Protein levels of TLR4, MyD88, and TRAF6. Mesenteric adipose tissue extracts were immunoblotted with anti-TLR4 (a), anti-MyD88 (b), and anti-TRAF6 (c). The results of scanning densitometry are expressed as arbitrary units. Bars represent means ± SEM of *n* = 6 mice, ^#^ *P* < 0.05 when compared to the high-fat diet and green tea (HG) group versus high-fat diet and water (HW) group. In (d), the representative bands of the molecules are shown. The membrane was stripped and immunoblotted with anti-*α*-tubulin antibody and used as loaded protein (lower panel in (d)).

The TLR4 protein levels in diet-induced obese mice (HW group) were significantly greater than chow-diet mice (CW group). Green tea treatment decreased this effect significantly (HG versus HW groups, *P* < 0.05; Figure 2(a)). The MyD88 protein levels in the HW group increased compared to the CW group. However, when obese mice were supplemented with green tea (HG group), this effect was attenuated (Figure 2(b)). The TRAF6 protein levels in diet-induced obese mice (HW group) were significantly greater than chow-diet mice (CW group). Green tea treatment significantly decreased this effect (HG versus HW groups, *P* < 0.05; Figure 2(c)).

## 4. Discussion

Numerous studies have been conducted to increase our understanding of the cause and treatment of obesity. In this sense, an alternative strategy is necessary such as phytotherapy treatment. Chronic systemic inflammation directly contributes to the development of obesity [27]. For instance, overweight and obese women generally have elevated serum levels of inflammatory cytokines, such as TNF-*α* [28, 29]. Therefore, suppressing chronic inflammation may be a good strategy to prevent and/or treat obesity. Interestingly, previous studies suggest the positive impacts of green tea polyphenols could be via its ability to suppress chronic inflammation [30, 31]. In addition, the impacts of green tea consumption on weight loss have been reported in clinical [32–36] and laboratory studies [37]. Antiobesity effects of green tea are probably due to its capacity to elevate thermogenesis and fat oxidation [38, 39]. Thus, we hypothesise that green tea supplementation reduces body-fat mass by regulating lipolytic pathway-related genes; such changes will result in downregulation of cytokine production and proinflammatory molecule protein levels. 

In the study, we measured body weight of the animals at the beginning and end of the study. Our results demonstrate that a high-fat diet induced body-weight gain (as observed by delta weight) and epididymal and mesenteric adipose tissue pads. However, green tea promoted a reduced delta weight and adipose tissue pads. Further, green tea extract led to increased lipolytic pathway protein levels, adiponectin, and anti-inflammatory cytokine IL-10 and reduced proinflammatory cytokine TNF-*α*.

The therapeutic uses of tea are confined to alternative medicine. Although the anticarcinogenic, anti-inflammatory, and antimicrobial properties of tea have been known for many years, clinical medicine has not included its use in treatments, almost certainly due to the lack of knowledge about its exact mechanisms of action [21, 22]. In human experiments, acute ingestion of green tea extract, which is mainly composed of catechins, has been reported to increase the proportion of whole-body fat utilisation by augmenting oxidation and lipolysis [23, 38, 39]. Lee et al. [40] demonstrated in an in vitro study that EGCG modulates the increase in lipolysis by directly increasing the gene expression of HSL, demonstrating its important role in lipid metabolism. Habitual consumption green tea extract has been reported to reduce body weight and body fat [32–36]; this may occur via increased lipolysis in adipose tissue, and our data support this.

The anti-inflammatory effect of green tea has been attributed to the polyphenol content [30, 31]. In Asian countries, green tea, which contains a class of polyphenols known as tea catechins, has been habitually consumed as one of the most popular beverages. Tea polyphenols have been shown to inhibit proteasome function, thereby terminating inflammation. Although tea polyphenols have been claimed to be the most potent constituents of tea, there is increasing evidence that these compounds are not the only constituents responsible for the beneficial effects on health from tea [41].

Our results demonstrate that green tea is able to decrease the protein content of TNF-*α* in adipose tissues and stimulate lipolytic enzymes. These conditions may favour reduced body weight and adipose tissues. In addition, we found that green tea reduced TLR4 expression, blocking proinflammatory effects. Youn et al. [42] showed that EGCG in cultured cells of the immune system had an anti-inflammatory effect, which was partly explained by the inhibition of the TLR. In summary, our results show that green tea extract intake increases expression of lipases, reduces adipose fat mass, and in parallel reduces inflammatory molecules and cytokines. Futures studies are needed to better understand the mechanism involved in the beneficial effects promoted by green tea extract intake, especially in mice fed a high-fat diet.

## Figures and Tables

**Table 1:** Composition of standard chow and high-fat diet.

Ingredients	Standard chow		High-fat diet	
	g/kg^−1^	Kcal/kg^−1^	g/kg^−1^	Kcal/kg^−1^
Cornstarch (Q.S.P.)	398	1590	116	462
Casein	200	800	200	800
Sucrose	100	400	100	400
Dextrinated starch	132	528	132	528
Lard	—	—	312	2808
Soybean oil	70	630	40	360
Cellulose	50	—	50	—
Mineral mix	35	—	35	—
Vitamin mix	10	—	10	—
L-Cysteine	3	—	3	—
Choline	2.5	—	2.5	—

Total	1000	3948	1000	5358

**Table 2:** Body weight and absolute and relative tissue weight.

Parameters	CW	CG	HW	HG
Initial weight (g)	27.9 ± 0.9	27.7 ± 1.2	28.3 ± 1.5	25.9 ± 0.6
Final weight (g)	45.4 ± 3.2	33.5 ± 1.5*	40.8 ± 1.3	33.9 ± 1.4^#^
Delta weight (g)	17.5 ± 1.3	5.8 ± 1.1*	12.5 ± 0.9	8.0 ± 0.7^#^
Epididymal (g)	0.81 ± 0.07	0.62 ± 0.09	1.45 ± 0.43^$^	0.52 ± 0.08^#^
Epididymal (%)	1.78 ± 0.16	1.85 ± 0.17	2.89 ± 1.12	1.41 ± 0.19^#^
Retroperitoneal (g)	0.44 ± 0.07	0.19 ± 0.03*	0.54 ± 0.08	0.39 ± 0.08
Retroperitoneal (%)	1.05 ± 0.21	0.57 ± 0.11*	1.35 ± 0.20	1.14 ± 0.22
Mesenteric (g)	0.45 ± 0.07	0.33 ± 0.06	0.86 ± 0.28^$^	0.23 ± 0.04^#^
Mesenteric (%)	0.96 ± 0.12	1.05 ± 0.23	2.16 ± 0.72^$^	0.65 ± 0.11^#^
Liver (g)	1.77 ± 0.09	1.55 ± 0.05	1.79 ± 0.18	1.37 ± 0.06
Liver (%)	3.99 ± 0.33	4.73 ± 0.30	4.42 ± 0.49	4.05 ± 0.17
Gastrocnemius (g)	0.19 ± 0.01	0.17 ± 0.02	0.21 ± 0.01	0.18 ± 0.01
Gastrocnemius (%)	0.43 ± 0.04	0.53 ± 0.09	0.51 ± 0.03	0.54 ± 0.05

*P < 0.05 chow diet and green tea (CG) group versus chow diet and water (CW) group (*n* = 12). ^$^ *P* < 0.05 high-fat diet and water (HW) group versus CW group (*n* = 12). ^#^ *P* < 0.05 high-fat diet and green tea (HW) group versus HW group (*n* = 12). g: grams; %: percentage.

**Table 3:** Serum concentrations of triacylglycerol (TAG), total cholesterol (TC), high-density lipoprotein (HDL), low-density lipoprotein (LDL), free fatty acids (FFA), and adiponectin in different experimental groups.

Parameters	CW	CG	HW	HG
TAG (mmol/L)	1.58 ± 0.04	1.57 ± 0.03	1.60 ± 0.04	1.46 ± 0.05
CT (mmol/L)	3.12 ± 0.08	3.29 ± 0.13	3.58 ± 0.17	3.54 ± 0.06
HDL (mmol/L)	1.54 ± 0.10	1.85 ± 0.16	1.81 ± 0.10	2.31 ± 0.13^#^
LDL (mmol/L)	0.83 ± 0.04	0.71 ± 0.06	1.11 ± 0.09^$^	0.87 ± 0.08
FFA (*μ*M)	1.21 ± 0.08	1.26 ± 0.08	1.02 ± 0.11	1.16 ± 0.13
Adiponectin (ng/mL)	82.25 ± 1.76	106.49 ± 2.91*	85.82 ± 2.53	112.02 ± 7.64^#^

*P < 0.05 chow diet and green tea (CG) group versus chow diet and water (CW) group (*n* = 12). ^$^ *P* < 0.05 high-fat diet and water (HW) group versus CW group (*n* = 12). ^#^ *P* < 0.05 high-fat diet and green tea (HW) group versus HW group (*n* = 12).

**Table 4:** Content cytokines in mesenteric adipose tissue.

Adipokines	CW	CG	HW	HG
Adiponectin (pg/*μ*g of protein)	0.24 ± 0.03	0.37 ± 0.02*	0.18 ± 0.01	0.40 ± 0.04^#^
IL-10 (pg/*μ*g of protein)	1.91 ± 0.26	11.27 ± 1.33*	3.47 ± 0.50	5.34 ± 0.47
TNF-*α* (pg/*μ*g of protein)	2.09 ± 0.79	1.92 ± 0.61	5.80 ± 0.47^$^	2.62 ± 0.61^#^

*P < 0.05 chow diet and green tea (CG) group versus chow diet and water (CW) group (*n* = 12). ^$^ *P* < 0.05 high-fat diet and water (HW) group versus CW group (*n* = 12). ^#^ *P* < 0.05 high-fat diet and green tea (HW) group versus HW group (*n* = 12).
